# Scientific echosounder data provide a predator’s view of Antarctic krill (*Euphausia superba*)

**DOI:** 10.1038/s41597-023-02187-y

**Published:** 2023-05-16

**Authors:** M. J. Cox, A. J. R. Smith, A. S. Brierley, J. M. Potts, S. Wotherspoon, A. Terauds

**Affiliations:** 1 Southern Ocean Ecosystem Program, Australian Antarctic Division, 203 Channel Highway, Kingston, Tasmania Australia; 2 Integrated Digital East Antarctica Program (IDEA), Australian Antarctic Division, 203 Channel Highway, Kingston, Tasmania Australia; 3 Australian Antarctic Program Partnership, Institute of Marine and Antarctic Studies, 20 Castray Esplanade, Battery Point, nipaluna / Hobart, TAS 7004 Australia; 4 Pelagic Ecology Research Group, School of Biology, Scottish Oceans Institute, Gatty Marine Laboratory, University of St Andrews, East Sands, St Andrews, Fife, St Andrews, KY16 8LB Scotland UK; 5 Secretariat of the Pacific Community, CPS – B.P. D5, 98848 Noumea, New Caledonia

**Keywords:** Ecosystem ecology, Marine biology, Behavioural ecology

## Abstract

Raw acoustic data were collected in East Antarctica from the RSV *Aurora Australis* during two surveys: the Krill Availability, Community Trophodynamics and AMISOR Surveys (KACTAS) and the Krill Acoustics and Oceanography Survey (KAOS) in the East Antarctic (centre coordinate 66.5° S, 63° E). The KACTAS survey was conducted between 14th to 21st January and 2001, and the KAOS survey was conducted between 16 January and 1 February 2003. We examine the Antarctic krill *(Euphausia superba*) component of these surveys and provide scientific echosounder (EK500 and EK60) data collected at 38, 120 and 200 kHz, cold water (−1 °C) echosounder calibration parameters and accompanying krill length frequency distributions obtained from trawl data. We processed the acoustic data to apply calibration values and remove noise. The processed data were used to isolate echoes arising from swarms of krill and to estimate metrics for each krill swarm, including internal density and individual swarm biomass. The krill swarm data provide insights to a predators’ views of krill distribution and density.

## Background & Summary

Management advice for fisheries targeting pelagic species often includes fishery-independent data gathered during active acoustic surveys with scientific echosounders^1^. Acoustic surveys of Antarctic krill (*Euphausia superba*) are often carried out from research vessels in order to estimate krill biomass^2^, which is used in conjunction with a population model to set catch limits^3,4^.

In 2001 the Krill Availability, Community Trophodynamics and AMISOR Surveys (KACTAS) voyage was conducted between 66°S and 67°S and 62°E to 64.5°E between 14th January and 21st January 2001. In 2003 the Krill Acoustics and Oceanography Survey (KAOS) survey was also conducted in the same area of the East Antarctic between 16 January to 1 February 2003. Both surveys aimed to measure krill distribution and abundance in an on-shelf region of the East Antarctic, and to examine the acoustic survey results in the context of oceanographic conditions^5^. Here we provide data from the fisheries acoustic components of the KACTAS and KAOS surveys that were collected using cold-water (water temperature −1 °C) calibrated EK500 and EK60 (Simrad, Horten, Norway) scientific echosounders operating at 38, 120 and 200 kHz.

Surveying in the Antarctic is logistically challenging, particularly in East Antarctic (longitude range 30°E to 150°E). Much of this region is only accessible by ship in the summer, and it can take at least seven days to get there from the closest port. In consequence, there are few data sets describing krill in this region, and data such as those presented here are an important resource for a range of research purposes.

Krill biomass estimates in a survey area are based on mean krill areal biomass density. Collapsing spatial information into a single estimate in this way does not provide information on how krill are distributed across the survey area. However, scientific echosounder data are high-resolution (e.g. 10 s m or 1 minute) so can reveal much about the distribution of krill. Krill are obligate schoolers, so are often found in swarms: in fact, swarms have been called the fundamental unit of krill biology^6^, so it seems reasonable that mapping the horizontal and vertical distribution of krill may reveal more about interactions between krill and their predators^7,8^ than would a simple two-dimensional map, that collapses depth. From a predator’s perspective, swarms represent an energy-rich resource, with a trade-off that swarms may be elusive and offer krill an important anti-predation advantage by engaging in collective predator-avoidance behaviour^9^.

## Methods

**Fig. 1:**
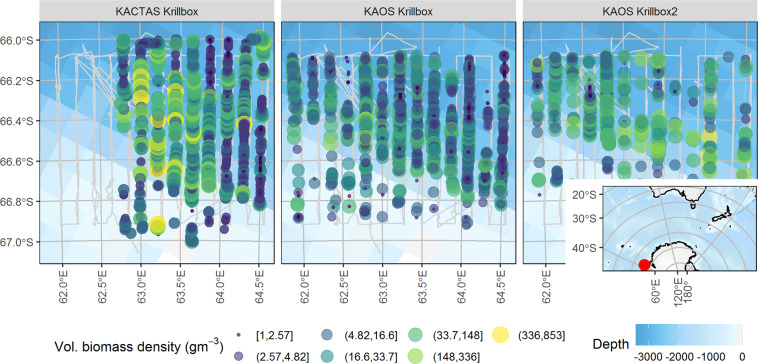
Survey location (solid red dot inset map) and the two surveys Krillbox (left hand panel), and Krillbox 2 (right hand panel). Each of the 13 transects are labeled and the vessel track is shown as a solid grey line. The internal volumetric density of each krill swarm (gm^−3^) is shown as coloured circles.

**Fig. 2:**
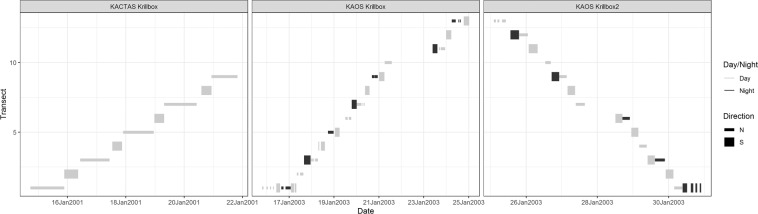
Survey effort in time. The left hand panel is the KACTAS survey (2001), with the centre and right hand panels the KAOS survey. There are two panels for the KAOS survey as there were two ‘Legs’ to the survey, i.e. one repeat of the survey. Time taken to sample a transect varies between transects because during KAOS some transects are sampled multiple times (denoted by Direction), or during KACTAS other sampling was conducted. During KAOS, acoustic sampling took place during day (light grey rectangles) and night (black rectangles).

The KACTAS and KAOS voyages, each consisting of 13 transects, the positions of which were defined formally pre survey, were conducted over the same area (Fig. 1). During KAOS two surveys were carried out, denoted as krill box 1 and krill box 2. The transects had a designed length of 90 km with a north-south orientation. Within each survey transects some transects were sampled multiple times: during the first survey 21 transects were sampled, and 18 transects in the second survey (Fig. 2). Sampling took place during day and night (Table 1).

**Table 1:** Sampling effort for the two surveys.

Survey	Number of transects				
	Total	Day	Night	Northerly direction	Southerly direction
Krillbox	21	14	7	11	10
Krillbox 2	18	13	5	9	9

The EK500 and EK60 scientific echosounders ran continuously during the surveys, operating at 38, 120 and 200 kHz. All echosounder transducers were hull-mounted (depth 5.5 m) split-beam transducers with 7° beam widths. Acoustic data were recorded to a range of 250 m at a 1 Hz ping repetition rate during the KACTAS voyage and 2 Hz pulse repetition rate during the KAOS voyage. The mean vessel speed was 7.8 knots giving a mean inter-ping spacing of 4 m for KACTAS and 2 m for KAOS.

In addition to the raw data that we describe here, we provide data on metrics of the krill swarms that were identified (e.g. Cox *et al*.^6^; Table 2). Krill swarms were extracted from data collected when the vessel was surveying along line transects, and not engaged in other activities, i.e. net sampling and CTD casts, or steaming between transects. Acoustic data were cleaned before krill swarm identification: seabed returns and surface noise were removed, as was background noise^10^. Seabed aliased returns were also removed manually. Potential krill swarms were delineated using the Shoal Analysis and Patch Estimation System (SHAPES) algorithm^11^ (implemented in Echoview v12.1 Echoview, Hobart Australia) was applied to the clean 120 kHz data convolved by a uniform 7 × 7 filter. The SHAPES algorithm parameters (Table 3) are identical to those of Tarling *et al*.^12^. The SHAPES algorithm identified potential swarm boundaries were applied to the 38 and 120 kHz clean data, and the mean volume backscattering strength was calculated within the boundaries for the 38 and 120 kHz data, denoted as *S*_*v*_*(38)* and *S*_*v*_*(120)*.

**Table 2:** Krill swarm metrics describing the position, morphometric and energetics.

Swarm metric name	Metric field names	Units	Description
Mean height	Height_mean	m	Mean swarm height
Mean depth	Depth_mean	m	Mean swarm depth
Start time stamp	Date_S, Time_S	YYYYMMDD HH:MM:SS.SSS	UTC date at start of swarm
Centre time stamp	Date_M, Time_M	YYYYMMDD HH:MM:SS.SSS	UTC date at centre (mid-point) of swarm
End time stamp	Date_M, Time_M	YYYYMMDD HH:MM:SS.SSS	UTC date at end of swarm
Position at start	Lat_S, Lon_S	dd.ddddd	Latitude and Longitude at start of swarm
Position at centre	Lat_M, Lon_M	dd.ddddd	Lat. and Longitude at centre of swarm
Position at end	Lat_E, Lon_E	dd.ddddd	Latitude and Longitude at end of swarm
Corrected swarm length	Length	m	Length of swarm corrected for beam geometry
Corrected swarm thickness	Thickness	m	Thickness of swarm corrected for beam geometry
Corrected perimeter	Perimeter	m	Length of detected edge of swarm corrected for beam geometry
Corrected area	Area	m^2^	Area of detected swarm (intersection between beam and swarm) corrected for beam geometry
Mean volume backscattering strength	Sv_mean_038 Sv_mean_120 Sv_mean_200	dB re 1 m^−1^	Calibrated data. NA denotes no signal at 200 kHz
Survey	Survey		Either the KACTAS or KAOS survey
Leg	Leg	—	Designates the first (krillbox) or repeat (krillbox 2) survey
Transect number	Transect		Arbitrary transect number
Pass	Pass	—	Sampling bout for a transect in a given direction.
Direction	Direction	—	Direction in which the transect was run either North (N) or South (S)
Day or night sampling	Light	—	Day or night sampling
Volumetric density	vol_den_gm3	gm^−3^	Krill swarm internal volumetric density
dB difference	dB120minus038	dB re 1 m^−1^	120 kHz-38 kHz Sv for a swarm
Swarm volume	volume_m3	m^3^	Assuming a cylindrical shape
Swarm biomass	biomass_g	g	Assuming a cylindrical shape

**Table 3:** Parameters for the Shoal Analysis and Patch Estimation System (SHAPES) algorithm.

Parameter	Value
Maximum horizontal linking distance (m)	15
Maximum vertical linking distance (m)	5
Minimum candidate length (m)	10
Minimum candidate height (m)	1
Minimum school length (m)	15
Minimum school height (m)	2
Minimum data threshold (dB re 1 m^−1^)	−70

The minimum data threshold of −70 dB re 1 m^−1^ is approximately one krill per m^3^.

Krill swarms were identified amongst all the potential swarms using the *S*_*v*_*(120) - S*_*v*_*(38)* ‘dB-difference’ approach e.g. Cox *et al*., Reiss *et al*.^6,13^. The ‘dB difference’ method uses the full version of the acoustic target strength model of krill, the Stochastic Distorted Wave Born Approximation (SDWBA; see Demer & Conti^14^, Calise & Skaret^15^), calculated at 1 mm length increments at 38 kHz and 120 kHz and the survey-specific length frequency distributions. The model parameters were those given in Calise & Skaret^15^ Table 1 except the distribution of orientations was ~Normal(mean = −28°, standard deviation = 20°) as used in Krafft *et al*.^2^ and Cox *et al*.^16^.

Krill length frequency distributions were sampled using a rectangular mid-water trawl (RMT 8 + 1^17^) during both KACTAS and KAOS surveys. Trawling was carried out in two modes: routine at locations predetermined stations, and target trawls which were carried out in response to krill-type echoes being detected by the echosounder (see Table 4 for net sample sizes, and minimum, mean and maximum krill lengths).

**Table 4:** Survey-specific net sampling details.

Survey	Number of krill sampled	Number of routine trawls	Number of target trawls	Minimum krill length (mm)	Mean krill length (mm)	Maximum krill length (mm)
KACTAS	8849	33	64	12.2	39.9	58.1
KAOS	9067	29	66	14.7	40.1	58.1

Following the method of Reiss *et al*.^13^, the dB difference range was calculated using the krill target strength and krill length frequency distribution observed during each survey. Using the acoustic target strength, a vector of dB differences was calculated for each of the 1 mm krill length increments (*l*), giving: *TS(120,l)-TS(38,l)*. The krill identification range was calculated using the dB difference at the minimum and maximum krill lengths (see Table 5 for survey specific krill identification dB difference ranges).

**Table 5:** Krill identification and scaling parameters.

Survey	Min. dB diff (dB)	Max. dB diff (dB)	Krill TS for scaling (dB re 1 kg)
KACTAS	1.0	16.2	−42.17
KAOS	1.0	14.8	−40.97

Krill identification parameters are from the ‘dB-difference’ method and are presented as the minimum (Min. dB diff) and maximum (Max. dB diff) values for the 120 kHz - 38 kHz frequency difference that were considered to be krill. Acoustic echoes from krill swarms were scaled from mean volume backscattering strength (S_v_, units: dB re 1 m^−1^) to volumetric density using the krill target strength (TS) of 1 kg (wetmass) of krill (units dB re 1 kg).

Krill swarm morphologies, i.e. length, perimeter and area, were corrected for beam geometry^18^. Following Diner^18^, schools with a relative dimension of less than two were deemed to be too small with respect to transducer beam geometry to have their morphology corrected, so were removed from further analysis.

The internal density of krill swarms, *ρ* [gm^−3^], was calculated following Cox *et al*., Brierley *et al*.^6,19^: *ρ*_*i*_ = *1000* × *10 exp{(S*_*vi*_*[120] - TS*_*kg*_*)/10}* where *S*_*v,i*_*[120]* is the mean volume backscattering strength (see^20^ for definition) at 120 kHz for the *i*th swarm and *TS*_*kg*_ is the target strength of 1 kg of krill at 120 kHz (Table 5).

The biomass [g] of the *i*th swarm was calculated by assuming swarms had a cylindrical shape, *β*_*i*_ = *π(h*_*i*_*/2)*^*2*^*l*_*i*_ *x ρ*_*i*_, where *h*_*i*_ is the average height of the *i*th swarm and *l*_*i*_ is the swarm corrected length. On average, the assumption of a cylindrical shape will underestimate swarm biomass as swarms are complex 3D shapes with larger volumes than cylinders and ellipses^21^.

## Data Records

### Processed acoustic and net data

The swarm data, krill length data, summary of transects, and the Echoview processing files are available at the AADC (dataset name AAS_4636_KACTAS_KAOS_KRILL) here doi:10.26179/39 × 3-9267.

### Raw acoustic data

If only a few raw acoustic data files are required, the files can be downloaded using the Australian Antarctic Data Centre (AADC) web browser here for KACTAS^22^:

https://data.aad.gov.au/datasets/science/AAD_Hydroacoustics_data/3_Data/2000-12_Aurora-Australis_KACTAS/Datasets/Echosounder/Simrad-EK500/Aurora-Australis/EK5and here for KAOS^23^:

https://data.aad.gov.au/datasets/science/AAD_Hydroacoustics_data/3_Data/2003-01_Aurora-Australis_KAOS/Datasets/Echosounder/Simrad-EK60/RAW/38H-120H-200H

The entire raw acoustic datasets for the KACTAS and KAOS surveys are 18.2 GB and 106.2 GB respectively and are available to download from a Simple Storage Service (S3) server. We provide instructions to download the entire KACTAS and KAOS datasets as a separate file called ‘Instructions for downloading the entire KACTAS and KAOS raw active acoustic data sets.pdf’^24^.

### Krill target strength data

The realization of the krill acoustic target strengths used in this study are available here:

https://github.com/ccamlr/2019Area48Survey/blob/09a26bb38d8eb231f9740367dd65413c05b5d781/results/SDWBA-TS-38-120-200.csv

### Oceanographic data

Whilst not the focus of this study, the oceanographic data of the KACTAS and KAOS surveys may be downloaded for the KACTAS survey^25^ and the KAOS survey^26^.

## Technical Validation

Echosounder performance is strongly dependent on water temperature (see for example^27,28^) making it important to calibrate echosounders in water at a similar temperature to that of the survey area. Here, the echosounders were calibrated in cold water (temperature −1 °C) for both KACTAS and KAOS and the calibration parameters (Table 6) were applied during data processing. The calibration was carried out using the standard methods of^29^ with a 38.1 mm diameter tungsten carbide sphere as the reference target. The calibration of the EK500 used during the KACTAS survey and the EK60 used during the KAOS survey were carried out in Horseshoe Harbour, Mawson on 24-Jan-2001 and 4-Feb-2003 respectively.

**Table 6:** Calibration parameters and settings for the EK500 scientific echosounder used during the 2001 KACTAS and the EK60 scientific echosounder used during the 2003 KAOS survey.

Survey	KACTAS			KAOS		
Instrument	EK500	EK500	EK500	EK60	EK60	EK60
Channel	T1	T3	T2	T1	T2	T3
Frequency (kHz)	38	120	200	38	120	200
Absorption Coefficient (dB m^−1^)	0.01	0.03	0.04	0.01	0.03	0.04
Absorption Coefficient Logging (dB m^−1^)	0.01	0.03	0.04	—	—	—
s_A_ correction (dB)				−0.53	−0.42	−0.44
Transducer gain (dB)	24.13	18.73	17.37	21.99	21.75	20.53
Transducer gain logging (dB)	23.52	18.73	17.37	—	—	—
S_v_ transducer gain (dB)	24.13	18.69	17.84	—	—	—
S_v_ transducer gain logging (dB)	23.52	18.69	17.84	—	—	—
Major Axis 3 dB beam angle (◦)	6.81	9.6	7.40	6.97	7.41	6.92
Major axis angle offset (◦)	−0.06	0	0	−0.09	0.02	−0.01
MinorAxis3dbBeamAngle (◦)	6.85	9.6	7.40	6.92	7.59	6.77
MinorAxisAngleOffset (◦)	−0.29	0	0	0.01	0.26	−0.03
Power (W)	2000	1000	1000	2000	1000	400
Pulse duration (ms)	1	1	1	1.024	1.024	1.024
Pulse duration logging (ms)	1	1	1	—	—	—
Two-way beam angle (dB re 1 sr)	−20.80	−18.00	−20.20	−20.6	−20.8	−20.8
Two-way beam angle logging (dB re 1 sr)	−20.80	−18.00	−20.20	—	—	—
Sound speed (ms^−1^)	1446	1446	1446	1446	1446	1446
Sound speed logging (ms^−1^)	1500	1500	1500	1500	1500	1500

## Usage Notes

The overall objective of the KACTAS and KAOS voyages was to clarify the relationship between the distribution of krill, predators of krill, and the surrounding oceanography. For example, the acoustic data from KAOS have also been used to compare krill predator (Adélie penguin, *Pygoscelis adeliae*)^5,30^ distribution to krill.

Since the main objective of the acoustic component of KACTAS and KAOS was to survey krill, the collection of high resolution acoustic data was facilitated by utilizing, a 1 Hz (KACTAS) and 2 Hz KAOS) pulse repetition rate, which was achieved by limiting the echosounder sampling range to 250 m. This was considerably less than the typical operational range achieved by 38 kHz echosounders of 600 to 1,000 m, e.g. Haris *et al*.^31^. Additionally, due to increased scattering at higher frequencies, 200 kHz returns were limited to 100 m depth. By using ping-based resampling (7 × 7 uniform convolution) during data processing, we have attempted to conduct schools-based analyses at a common scale. Nevertheless, given the different ping rates between the two surveys, researchers should be mindful of the inherent difference in the underlying along-transect (horizontal) spatial resolution of the two data sets.

Also, as krill were observed to 250 m, we investigated the number of swarms that may have resided below 250 m and remained undetected. Using log-normal statistical distributions fitted to the vertical distribution of krill for each survey, we estimate only 0.2% of krill swarms were found below 250 m during the KACTAS survey and 0.5% during the KAOS survey.

**Fig. 3:**
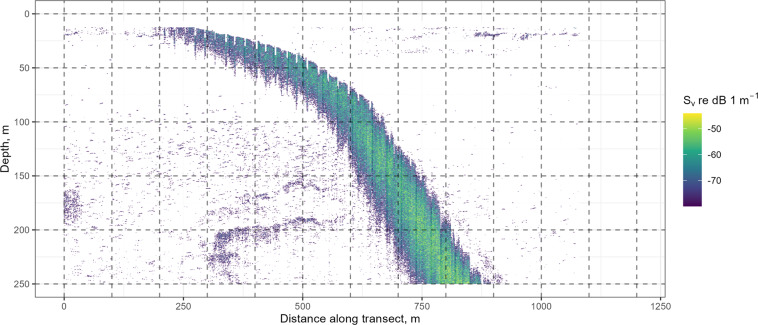
Example seabed aliased echoes shown as a mean volume backscattering strength (S_v_) echogram from the EK60 38 kHz echosounder used during the KAOS survey.

Due to rapidly changing bathymetry in some parts of the survey area, there were numerous instances of seabed aliased returns (false bottom) in the 38 kHz (Fig. 3) record, and occasionally the 120 kHz record. Methods for real-time removal of seabed aliased returns, such as Renfree & Demer^32^ were not available at the time of these surveys, and automated post processing methods are not yet readily available (but see Blackwell *et al*.^33^), so aliased seabed returns had to be removed manually before school detection took place. The aliased seabed returns are available as Echoview regions (.EVR) files, but if other acoustic processing software is used, the aliased seabed returns must be removed before schools detection or echo integration.

The echosounders were run continuously during the KACTAS and KAOS voyages including during CTD and net sampling, so these data should be removed if the objective of future analysis is based on line transect sampling. Also, compared to line transect sampling, the CTD and net sampling regions were subject to additional sources of noise, e.g winch and propulsion.

The echosounder calibration values given here (Table 6) present the best available estimates obtained from the available data (digital and field notebooks) using the calibration field procedures and software available at the time of the surveys. Whilst the calibration method was published and well understood before both the KACTAS and KAOS surveys, it wasn’t until 2015 the fisheries acoustics community published their best-practice recommendations for echosounder calibration (Demer *et al*.^29^). The calibration values (Table 6) may not have been obtained or processed in line with current best practice. For example, whilst in line with best-practice at the time of the surveys, subsequent research has shown that lower power settings are required to avoid non-linear effects^34^, so we recommend the raw acoustic data are not reprocessed for krill biomass estimation.

Both KACTAS and KAOS surveys were carried out more than 20 years ago so echosounder data logging settings were in part selected due to data storage constraints and available echosounder file format options. As with many acoustic surveys of this time, the KACTAS acoustic survey data were collected using a Simrad EK500 scientific echosounder with data written in the .EK5 format (or Q-telegram) to reduce file size. Whilst the .EK5 format is not used by the next generation of echosounders, it was the best-available data format at the time of the survey and we are not aware of any conversion tools to change .EK5 data to other formats.

## Data Availability

The EchoviewR package^35^ that was used to automate the Echoview processing is available from a Github public repository: https://github.com/AustralianAntarcticDivision/EchoviewR.
